# Insights for the Captive Management of South China Tigers Based on a Large-Scale Genetic Survey

**DOI:** 10.3390/genes15040398

**Published:** 2024-03-24

**Authors:** Wenping Zhang, Kaixiong Lin, Wenyuan Fu, Junjin Xie, Xueyang Fan, Mingchun Zhang, Hongxing Luo, Yuzhong Yin, Qiang Guo, He Huang, Tengteng Chen, Xipan Lin, Yaohua Yuan, Cheng Huang, Shizhang Du

**Affiliations:** 1 Key Laboratory of Monitoring Biological Diversity in Minshan Mountain of National Park of Giant Pandas, College of Life Science & Biotechnology, Mianyang Normal University, Mianyang 621000, China; zhang_zoology@163.com (W.Z.);; 2 Fujian Meihuashan Institute of South China Tiger Breeding, Longyan 364201, China; mhslkx@163.com (K.L.); 19500141957@163.com (H.L.);; 3 Longyan Geopark Protection and Development Center, Longyan 364201, China; 4 Chengdu Research Base of Giant Panda Breeding, Chengdu 610081, China; 5 China Conservation and Research Center for the Giant Panda, Chengdu 611830, China; nbaliker2@163.com; 6 Chongqing Zoo, Chongqing 400050, China; 7 Shanghai Zoo, Shanghai 200335, China

**Keywords:** South China tiger, genetic rescue, hybrid, genome

## Abstract

There is an urgent need to find a way to improve the genetic diversity of captive South China tiger (SCT, *Panthera tigris amoyensis*), the most critically endangered taxon of living tigers, facing inbreeding depression. The genomes showed that 13 hybrid SCTs from Meihuashan were divided into two groups; one group included three individuals who had a closer relationship with pureblood SCTs than another group. The three individuals shared more that 40% of their genome with pureblood SCTs and might be potential individuals for genetic rescuing in SCTs. A large-scale genetic survey based on 319 pureblood SCTs showed that the mean microsatellite inbreeding coefficient of pureblood SCTs decreased significantly from 0.1789 to 0.0600 (*p* = 0.000009) and the ratio of heterozygous loci increased significantly from 38.5% to 43.2% (*p* = 0.02) after one individual of the Chongqing line joined the Suzhou line and began to breed in the mid-1980s, which is a reason why the current SCTs keep a moderate level of microsatellite heterozygosity and nucleotide diversity. However, it is important to establish a back-up population based on the three individuals through introducing one pureblood SCT into the back-up population every year. The back-up population should be an important reserve in case the pureblood SCTs are in danger in the future.

## 1. Introduction

Tigers (*P. tigris*) play important ecological roles in the ecosystems where they occur and are powerful cultural symbols [1]. At present, six extant tiger subspecies have been proved through whole-genome sequencing analyses from 32 voucher specimens [2] by Liu et al. [3] and Sun et al. [4]. Tidière et al. [5] revealed that the six subspecies presented different reproductive and survival patterns. Among the six subspecies, the Sumatran tiger is the only island population with significant genetic distances from all continental tigers [6] and the South China tiger is the rarest tiger subspecies (https://www.iucnredlist.org) (accessed on 15 December 2021). Thus, these results show that the six tiger subspecies should be managed separately in order to avoid hybridization.

The fossil records and genetic analyses of tigers both show that South China tigers (SCT) play a very important role in tiger evolution [1,2,3,7], which was proved via ancient DNA. A genome-wide monophyly of ancient DNA supported the SCT as a distinct subspecies; eastern China was a genetic melting pot that fostered divergent lineages to merge into the SCT and other subsequent northern subspecies to develop [4]. In history, the SCTs maintained a broad distribution across many biomes from forests to deserts; however, in recent decades the range has been significantly condensed [1]. A survey of SCTs from 2001 to 2002 found no evidence of this unique subspecies of China in the wild [8]. SCT was, therefore, believed to be “functionally extinct” in the wild and captive SCT populations may constitute the last hope for the conservation of this endangered tiger subspecies. Moreover, due to the SCT’s great importance as a top predator in the ecosystem, the Chinese government is actively promoting its reintroduction into the wild [9]. The first step of the reintroduction is to check the genetic diversity of the captive SCT population.

The captive SCTs are descended from six individuals captured from the field from 1950s to 1970s; they form two lines: the “Chongqing line” and the “Suzhou line” [2,10,11]. At present, 248 individuals are living in 17 institutions (16 zoos in China and 1 institution in South Africa) in October 2023. Pedigree-based data show the current captive SCT population is severely inbred with an increase in the pedigree inbreeding coefficient from 0.2586 in 1999 to 0.3584 in 2016; only 65.23% of six ancestors’ genetic diversity exists in the population [12]. Following the whole-genomic data of tigers, Zhang et al. [13] found 43 long runs of homozygosity fragments that were shared by 15 individuals in the SCT population that covered a total length of 20.63% in the SCT genome. Consistent with the results of Zhang et al. [13], Wang et al. [14] also found that the SCT had high genomic inbreeding values for longer runs of homozygosity (ROH > 1 Mb). Zhang et al. [13] found a lower nucleotide diversity (π) in the SCT population than that in the Amur tiger population. However, the genetic diversity of the SCT based on genome data was not as low as what was inferred from its pedigree records. The genomic heterozygosity of the SCT was moderate among the six subspecies [14], which is consistent with that of Zhang et al. [10] who found that captive SCTs kept moderate levels of genetic variability following microsatellite loci data, which was slightly lower than that in the captive Amur tiger population [15]. Hybridization between the South China tiger and other tiger subspecies is believed to have occurred in some Chinese zoos [16], which is supported by genetic characterization using mitochondrial and microsatellite DNA markers [10]. Wang et al. [14] also identified some SCTs harbored some rare genetic variants introgressed from other tiger subspecies, which may be the reason why the SCT population maintained a moderate genetic diversity. In order to avoid the impact of hybridization on the SCT population, it is necessary to conduct more analysis using pureblood SCTs.

In addition, the captive SCT population is facing other difficulties, such as juvenile survivorship and fecundity [17], the highest cub mortality rate as well as the smallest litter size among tiger subspecies [5], high juvenile and especially neonatal mortality [18], and impaired adult fertility [12,18]. Moreover, the population is facing the risk of declining; the number of alive pureblood SCTs decreased by 10 from 2022 with 246 to 2023 with 236 [11]. These difficulties might be due to the inbreeding depression among SCTs [5,12,18] and could not be avoid via careful genetic management. Genetic diversity is central to conservation efforts and plays an important role in considering to what extent captive breeding programs prioritize it [19,20]. When a small number of animals are left for breeding, genetic drift in the small population becomes stronger and inbreeding increases, which can reduce fitness; thus, inbreeding depression is a big problem in small populations [21,22]. So, it is necessary to find a way to improve the genetic diversity of captive SCTs.

In this study, we focused on one main scientific question: how to manage and improve the genetic diversity of captive SCTs? In order to solve this question, we characterized the inbreeding coefficient of the captive SCT population. Then, we combined the microsatellite and whole-genome data and analyzed the genomic difference and deleterious mutation load of a hybrid population from Meihuashan. These findings do not only explain the successful breeding history of the captive SCTs but also pave the way for how to manage hybridization in order to increase genetic diversity and decrease the extinction risk in the SCT population in the future.

## 2. Materials and Methods

### 2.1. Samples

In order to avoid hybridization in the SCT population, the Committee of TAG of SCT has requested all institutions with SCTs to send blood samples of every SCT to our lab for paternity identification every year since 2012. From this sample set, 211 alive pureblood SCTs were sampled, representing approximately 86% of the 246 alive pureblood SCTs listed in the International South China Tiger Studbook of 2022 [11] (Table 1 and Table S1). Moreover, the samples from 110 dead pureblood SCTs were also included in this study (Table S1). In addition, the microsatellite data of 108 voucher tigers [2,15] were used as the reference tiger population data set, just like in the method of Zhang et al. [10]. 

Generational overlap is normal for captive-born animals and a generation in SCTs is calculated as the average generation of the parents plus 1, following the formula (G0 + G1)/2 + 1 where G0 and G1 are the generations of its father and mother, respectively, following the method of Farquharson et al. [23].

The pedigree inbreeding coefficient (*f*_P_), ancestral inbreeding coefficient (*f*_a_Kal_), and new inbreeding coefficient (*f*_New_) according to Kalinowski et al. [24] were estimated using the GRAIN package, version 2.2 [25].

### 2.2. Mitochondrial DNA Analysis

The cytoplasmic mitochondrial DNA sequences used in Luo et al. [2] and Zhang et al. [10] were chosen in our research. After the PCR products were amplified and purified, the products were processed using an ABI 310 DNA sequencer. The sequences were unambiguously aligned using BioEdit and visually inspected.

### 2.3. Microsatellite Analysis

Thirty polymorphic microsatellite loci were used to analyze the genetic structure of captive SCTs with voucher tiger genetic profiles under previously published conditions [2,10]. MICROCHECKER v. 2.2.3 [26] was used to check the quality of microsatellite genotypes which included possible null alleles, allele dropout, and scoring errors. Microsatellite genetic variation, including the average heterozygosity, average number of alleles per locus, number of unique alleles, and average variance, was obtained following MICROSAT [27].

A Bayesian-model-based clustering approach with a series of independent runs was performed using population clusters (*K*) from 3 to 19, assuming an admixture model, with burn-in and replication values set at 50,000 and 10^6^, respectively, and with Usepopinfo = 1 in STRUCTURE 2.3 [28] following the method of Zhang et al. [10]. The individual inbreeding coefficient of microsatellite data (*f_M_*) reflects the extent to which their parents are genetically related; thus, the triadic maximum likelihood (TrioML) estimator [29] implemented in Coancestry [30] was used to estimate *f_M_* for each tiger based on microsatellite data. According to the conventional cut-off standard, *f_M_* < 0.125 is defined as low inbreeding, 0.25 > *f_M_* ≥ 0.125 as moderate, and *f_M_* ≥ 0.25 as high [31].

### 2.4. Genome Analysis

Following the above microsatellite data, 23 pureblood and 13 hybrid SCTs were screened to analyze genetic diversity at the genome level (Table 1 and Table S1). Out of the 36 samples, 21 voucher specimens came from Wang et al. [14]; the remaining 15 SCTs were sampled bloods (Table S1) used to extract DNA using the DNeasy Blood and Tissue Kit (QIAGEN, Valencia, California, USA) following the manufacturer’s protocols. One 350 bp sized DNA library was constructed and sequenced using the Illumina Hiseq X Ten platform for 150 bp paired-end reads following the pipeline of the Genome Center of Novo Genomics (Tianjin, China). In addition, we obtained the 32 published genomes of 6 tiger subspecies from Liu et al. [3] (Table S1). The genome of the domestic cat in Wang et al. [14] was used as the outgroup for the phylogenetic construction. The genome data sets are available from the CNGB Sequence Archive (CNSA) of China National GeneBank DataBase (CNGBdb) with accession number CNP0005449.

The above 69 genomes were analyzed according to the same pipeline as the following. After a series of quality control (QC) procedures of re-sequencing following the pipeline of Wang et al. [14], the clean reads of each sample were then mapped to the SCT genome (AmyTig1.0) [14] using bwa-mem (version 0.7.17) [32] with default parameters. Alignment files were converted to BAM files using SAMtools (settings: -bS -t) (v-0.1.19) [33]. After removing potential PCR duplications using Picard (version 2.25.6) (https://github.com/broadinstitute/picard/releases/tag/2.25.6) (accessed on 20 March 2023), the Genome Analysis Toolkit (GATK) package [34] was used to call SNPs following the filtering criteria of Wang et al. [14]. At last, a total of 11,458,929 high-quality SNPs from tigers were retained for subsequent analyses.

Three methods (PCA, NJ, and ADMIXTURE) were used to evaluate the genetic relationship between pureblood and hybrid SCTs. Principal component analysis (PCA) was carried out based on the 11,458,929 SNPs by using the --pca function in PLINK 1.9 (settings: --bfle, --pca –noweb) [35]. PCA results were visualized using R (version 3.6.3). An NJ tree based on a *p*-distance matrix using VCF2Dis (https://github.com/BGI-shenzhen/VCF2Dis) (accessed on 22 March 2023) was generated using FastMe2.0 (http://www.atgc-montpellier.fr/fastme/) (accessed on 22 March 2023) and visualized using iTOL [36]. We further reduced the number of variants by removing nonbiallelic and missing markers, filtering using MAF < 0.05, and LD-pruning using PLINK version 1.07 [35], with the following parameters: --geno 0.05 --maf 0.05 --hwe 0.0001 --ld-window 999,999 --ld-window-kb 10 --ld-window-r2 0.2 --r2 --make-bed. The final data set used for the ADMIXTURE contained 64,653 SNPs. To assess the variability in the estimates obtained via ADMIXTURE, the program was run 3 times for the genomic data set, with the number of groups (*K*) varying between 2 and 14, and a fivefold cross-validation [37].

The software vcftools (version 0.1.13) [38] was used for genome-wide genetic diversity estimation using a sliding-window approach (20 kb windows sliding in 10 kb steps). The genomic heterozygosity of individuals was calculated using the method-of-moments implemented in the --het function in PLINK 1.9 [35]. The runs of homozygosity (ROH) may reflect historical population homozygosity by descent and long ROHs are probably the result of recent inbreeding [39,40]. Thus, an analysis of ROH was estimated via PLINK (v1.9) with parameters from Wang et al. [14]. *F*_ROH_, which is an estimate of ROH proportion in an individual genome [41], was obtained via the pipeline of Zhang et al. [13].

Deleterious nonsynonymous SNPs of individuals can be used to explore the potential influence in populations. ANNOVAR [42] was used for gene-based SNP annotation. Genetic variant annotation and functional effect prediction in nonsynonymous SNPs were analyzed using SnpEf (5.2) [43] following the procedures of Zhang et al. [13].

### 2.5. Statistical Analysis

The correlations among the pedigree inbreeding coefficient, genome-wide heterozygosity, microsatellite heterozygosity, *F*_ROH_, and generations were plotted and estimated using R (ggscatter (cor.coef = TRUE, cor.method = ‘Pearson’) from the ggpubr R package) (https://rpkgs.datanovia.com/ggpubr/) (accessed on 25 March 2023). A Wilcoxon test (compare_means (method = ‘wilcox.test’, p.adjust.method = ‘BH’) from the ggpubr R package was used to test the significance of the difference between groups.

## 3. Results

### 3.1. The Identification of Pureblood SCTs with Mitochondrial DNA and Microsatellite

Following the results of Zhang et al. [10], the hybrid individuals in the SCT population and their offspring were excluded and a total of 320 pureblood SCTs were obtained for mitochondrial DNA and microsatellite analysis (Table S1). Two concatenated mtDNA haplotypes were detected from the 320 pureblood SCTs, corresponding to voucher subspecies haplotypes AMO1 (N = 266, 83% of the sampled captive population) and COR1 (N = 54, 17% of the sampled captive population) (Table S1), which showed AMO1 constituted the majority of mitochondrial haplotypes in the SCT population.

**Figure 1:**
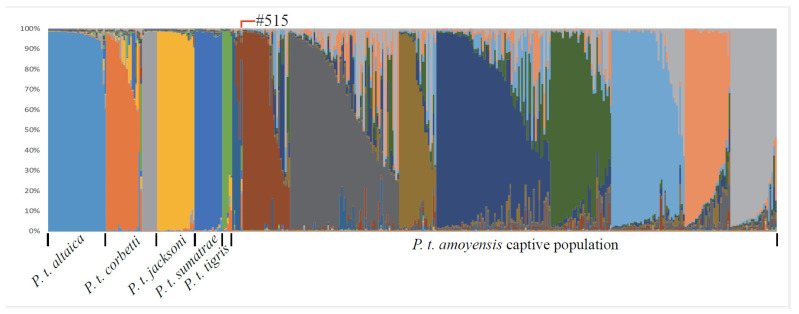
The results of assignment of individuals following the microsatellite genotype of 30 loci. Here, the population structure when K = 15, which produced the highest probability among other choices of K (Figure S1), and each individual were represented by a thin vertical bar. SCTs did not include the individuals and their offspring with an admixture of other tiger subspecies identified by Zhang et al. [10]. A total of 320 SCTs were included in this figure. #515 was identified as an admixture of other tiger subspecies.

The genotypes of the 320 SCTs samples were combined with 108 published voucher tigers [2,15] and applied to STRUCTURE analysis. The highest log likelihood value of the data (Ln probability) was obtained when K = 15 (Figure S1) and the 108 voucher tigers exhibited similar genetic structures as described by Luo et al. [2] (Figure 1). The 320 SCTs exhibited distinct genetic structures from other tiger subspecies and were further divided into nine clusters (Figure 1). A high percentage (a total of 42.3%) of genetic composition of other tiger subspecies, especially Indochinese tiger (17.1%), was observed in #515 (Figure 1; Table S2). The *International South China Tiger Studbook* [11] shows #515 is the offspring of #392 and #421. However, the father-mother-offspring of #421-#392-#515 was excluded by parentage verification with microsatellite genotype discrepancies identified between the three individuals (Table S2). As a result, subsequent exclusion of #515 and its offspring from the captive South China tiger breeding program is recommended. So, a total of 319 pureblood SCTs were included in this study. Out of the 319 tigers, 210 tigers were living before 31 December 2022.

A total of 117 alleles were found in the 319 pureblood SCTs and 13 alleles (11%) were lost from the living 210 pureblood SCTs, which included FCA043-119, FCA44-112, FCA069-105, FCA069-109, FCA077-134, FCA091-134, FCA094-210, FCA123-150, FCA126-146, FCA161-175, FCA229-158, FCA290-226, and FCA304-121; one locus (FCA229) had just one allele in the living 210 SCTs. In addition, 17 alleles were found to be individual-specific alleles in the 210 SCTs. The levels of microsatellite genetic diversity in the living 210 SCTs showed the average number of alleles per microsatellite locus was 3.5517 and the mean microsatellite variance was 7.6904. The 210 SCTs harbored a moderate level of microsatellite heterozygosity with 0.4205.

### 3.2. The Inbreeding of Pureblood SCTs

In order to detect inbreeding in the 319 pureblood SCTs (Figure 1; Table S1), *f_M_* (individual inbreeding coefficient of microsatellite data) and *f*_P_ (individual inbreeding coefficient of pedigree data) were obtained. The *f*_P_ data of the 319 SCTs show that the mean *f*_P_ is 0.336 with a maximum of 0.492. The mean *f*_New_ is 0.284 with a maximum of 0.407 and the mean *f*_a_Kal_ is 0.053 with a maximum of 0.184. Comparisons between *f*_a_Kal_ and *f*_New_ showed an average relative contribution of 84% of new inbreeding to individual inbreeding. A lower value for *f*_M_ than for *f*_P_ occurred among the 319 SCTs with a mean of 0.067 and a maximum of 0.6183 for *f*_M_. The Pearson correlation showed that a lack of correlation was found between *f_M_* and *f*_P_ (r = 0.099, *p* = 0.078; Figure S2). The *f*_M_ showed that 260 SCTs (82%) had low inbreeding (<0.125), 39 SCTs (12%) had moderate inbreeding (0.25 > *f*_M_ ≥ 0.125), and only 20 SCTs (6%) had high inbreeding (≥0.25), following the standards of Marshall et al. [31].

In order to detect whether the generation plays a role in inbreeding in SCTs or not, we analyzed the correlation between *f_M_*, *f*_P_, and the heterozygous loci ratio with generation. The Pearson correlation showed a very weak negative correlation between the heterozygous loci ratio and *f*_P_ (r = −0.147, *p* = 0.009; Figure S3) and a strong negative correlation between the heterozygous loci ratio and *f*_M_ (r = −0.686, *p* < 0.0001; Figure S4). The results also showed a very weak correlation between *f*_P_ and generation (r = 0.195, *p* < 0.001; Figure S5) and between *f*_M_ and generation (r = −0.280, *p* < 0.0001; Figure S6), but a lack of correlation was found between the heterozygous loci ratio and generation (r = −0.078, *p* = 0.17; Figure S7).

**Figure 2:**
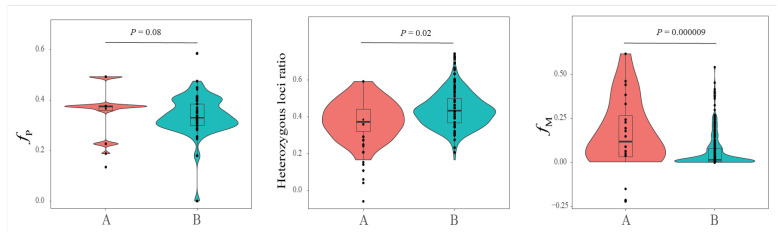
The distributions of estimated individual inbreeding coefficients (*f*_P_ and *f*_M_) and the heterozygous loci ratio based on microsatellite data. Here, A indicates the group including the individuals without the genetical materials of #140 and B indicates the group including the individuals with the genetical materials of #140. The *p*-value was obtained from an ANOVA test. *f*_P_: pedigree inbreeding coefficient, *f*_M_: microsatellite inbreeding coefficient.

Following the *International South China Tiger Studbook* [11], just one individual #140 belongs to pureblood Chongqing line without admix genetics of the Suzhou line [10]. We compared the difference in the inbreeding coefficient and the heterozygous loci ratio between individuals with and without the genetics of #140. Out of the 319 pureblood SCTs, just 20 individuals did not include the genetical materials of #140. The inbreeding coefficient showed the 20 individuals had high inbreeding with mean *f*_P_ = 0.3652 (maximum *f*_P_ = 0.4921) and mean *f*_M_ = 0.1789 (maximum *f*_M_ = 0.6183) and had a very low heterozygous loci ratio with 38.5% as the mean and 16.7% as the minimum. When #140 joined in SCTs, the population of pureblood SCTs significantly decreased inbreeding and the increased heterozygous loci ratio (Figure 2). After #140 joined in SCTs, the mean *f*_P_ decreased from 0.3652 to 0.3344 without a significant difference (*p* = 0.08), the mean *f*_M_ decreased significantly from 0.1789 to 0.0600 (*p* = 0.000009), and the ratio of heterozygous loci increased significantly from 38.5% to 43.2% (*p* = 0.02) (Figure 2).

The inbreeding values of the living 210 pureblood SCTs show that 86.2% (181) of the 210 sampled individuals have a low inbreeding coefficient of *f*_M_ < 0.125, 11.0% of the individuals (23 out of 210) have a moderate inbreeding coefficient with 0.25 > *f*_M_ ≥ 0.125, and only 2.8% have a high inbreeding coefficient of *f*_M_ ≥ 0.25. Eleven institutes with more than three living pureblood SCTs (Table 1) were used to compare the differences in their inbreeding coefficients and the results showed no significant differences among institutes, although Suchou Zoological Garden had maximum mean inbreeding coefficient of 0.08 (Figure S8).

### 3.3. The Genomic Diversity of Pureblood SCTs

**Figure 3:**
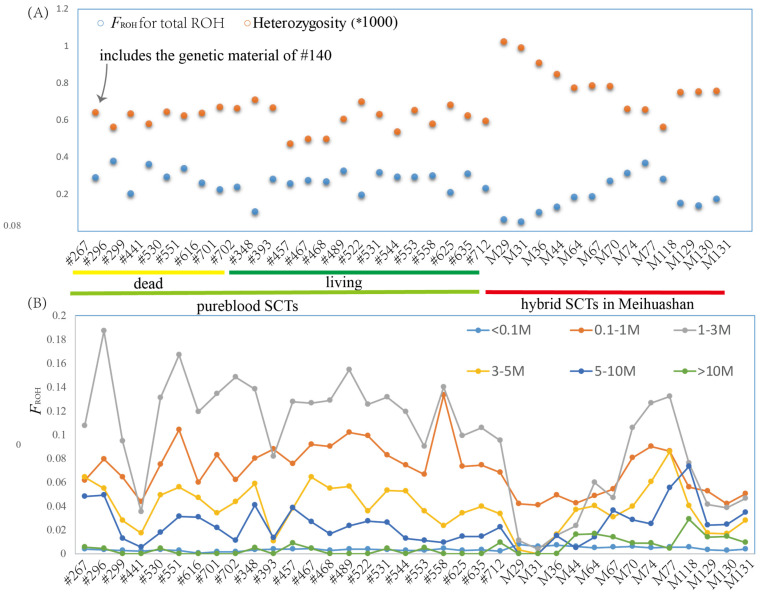
Distribution of individual heterozygosity and genomic inbreeding coefficients (*F*_ROH_). (**A**) Genome-wide heterozygosity and *F*_ROH_ per individual. (**B**) *F*_ROH_ based on different lengths of runs of homozygosity (ROH), with a minimum length of 100 kb, per individual.

A total of 23 pureblood SCTs had genome data (Table S1) and their covered ratio ranged from 98.21% to 99.10% (Tables S3 and S4). The SNP density (number of variants per kb) averaged 0.91 ± 0.023 of the pureblood SCT population. A strong positive correlation occurred between genome-wide heterozygosity, measured as heterozygous positions per base pair of the callable genome, and nucleotide diversity (π) (r = 0.982, *p* = 2.22312 × 10^−26^; Figure S9). The nucleotide diversity (π) of the pureblood SCT population ranged from 2.0 × 10^−7^ to 0.0017 (mean = 0.00029 ± 6.62589 × 10^−6^); their genome-wide heterozygosity averaged 0.00061 ± 1.35243 × 10^−5^. No correlation was found between generations and genome-wide heterozygosity or nucleotide diversity (π). Among the 23 individuals with genomes, #267 with the genetic material of #140 does not have significantly higher or lower nucleotide diversity and heterozygosity than other individuals with pureblood SCT genetic materials (Figure 3A); no significant difference was found between dead and alive pureblood SCTs (*p* > 0.05) (Figure 3A).

Individual inbreeding based on genome-wide SNPs using the inbreeding coefficient *F*_ROH_ (based on the runs of homozygosity (ROH) ≥ 10 kb) [40] showed that a total of 17,307 ROH was identified among the 23 pureblood SCTs, with an average number of 779.44 ± 34.71 that ranged from 10092 bp to 12.75 Mb in physical length (Figure 3 and Table S5). A strong negative correlation was found between heterozygosity and *F*_ROH_ for total ROH (r = −0.775, *p* = 2.83221 × 10^−8^; Figure S10) (Figure 3A). Similar to heterozygosity, the individual #267 does not have a significantly higher or lower *F*_ROH_ for total ROH than other individuals with pureblood SCT genetic materials (Figure 3A). However, individual #267 had the longest ROH with 12.75 Mp among the 23 pureblood SCTs (Table S5). In addition, among the twenty-three pureblood SCTs, seven tigers, including #267, #296, #489, #530, #558, #625, and #712, all had long ROH values (>10 Mb). Individual #348 had the lowest *F*_ROH_ for total ROH and did not have any long ROH occurrences (>10 Mb) (Figure 3, Table S5), suggesting #348 to be the least inbred individual. No significant difference was found between dead and living pureblood SCTs for *F*_ROH_ under total ROH and different lengths of ROH (*p* > 0.05) (Figure 3).

### 3.4. The Genomic Diversity of Hybrid SCTs

As expect, the 13 hybrid SCTs from Meihuashan had high genetic diversity; their SNP density (mean = 1.15 ± 0.03), nucleotide diversity (π) (mean = 0.00037 ± 1.64061 × 10^−5^), and genome-wide heterozygosity (mean = 0.00079 ± 3.61349 × 10^−5^) all were higher than those in the pureblood SCT population (*p* < 0.001) (Table S4 and Figure 3A). The *F*_ROH_ in total ROH was significantly lower in the hybrid SCTs than the pureblood SCTs (*p* = 0.0026, ANOVA) (Table S4, Figure 3A and Figure S11), which shows the hybrid SCTs in Meihuashan have lower inbreeding than the pureblood SCTs. However, the differences in *F*_ROH_ between the hybrid and pureblood SCTs depended on different ROH lengths (Figure 3B and Figure S12): the hybrids had lower *F*_ROH_ for 0.1 M ≤ the ROH length < 3 M than purebloods (*p* < 0.01, ANOVA) (Figure 3B and Figure S12B,C), the hybrids had higher *F*_ROH_ than purebloods (*p* < 0.01, ANOVA) for longer ROH lengths (>10 M) or shorter ROH lengths (<0.1 M) (Figure 3B and Figure S12A,F), and the difference between purebloods and hybrids SCTs was not significant (*p* > 0.05, ANOVA) for 3M ≤ the ROH length < 10 M (Figure 3B and Figure S12D,E).

### 3.5. The Genomic Difference between Pureblood and Hybrid SCTs

**Figure 4:**
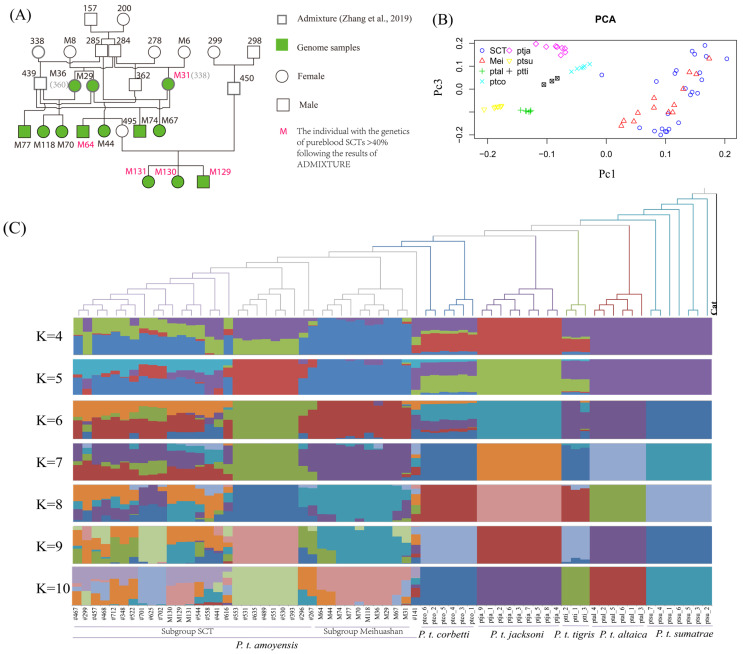
Population genomic structure of tiger subspecies following autosomal variants. (**A**) Family tree of the 13 hybrid SCTs in Meihuashan are shown here. The number in the family tree is the studbook number or the code in Meihuashan. (**B**) Principal component analysis (PCA) of PC1 and PC3 indicates six distinct clusters corresponding to current subspecies designations and South China tigers including SCT and Mei. SCT denotes the 23 pureblood SCTs and Mei denotes the hybrid SCTs in Meihuashan. ptal: *P. t. altaica*, ptco: *P. t. corbetti*, ptja: *P. t. jacksoni*, ptsu: *P. t. sumatrae*, and ptti: *P. t. tigris*. (**C**) Population genetic structuring of different tiger subspecies inferred from the phylogenetic relationship and ADMIXTURE [37]. The phylogenetic tree was constructed using the domestic cat as an outgroup and all nodes are of 100% reliability. Each individual is represented by a thin vertical bar, which are partitioned into K colored segments and represent the individual affiliation to each cluster (K is set from 4 to 10).

The results of PCA using genome-wide data showed that five living tiger subspecies, except *P. t. jacksoni*, were separated along the first principal component (PC1, Figure 4B), while the pureblood SCTs (SCT) and the hybrid SCTs (Mei) were separated along PC2, which could not separate *P. t. jacksoni* from other tiger subspecies (Figure S11). The pureblood and hybrid SCTs clustered together when PC1 combined with PC3, with a clearly separation of the living six tiger subspecies (Figure 4B), which was supported by phylogenetic relationship and ADMIXTURE (Figure 4C).

The NJ tree, based on pairwise genetic distances using the domestic cat as an outgroup, supports the taxonomic status of six distinct tiger subspecies [2,3,4,14], which was confirmed by the admixture analysis (Figure 4C). From K = 4 to 10, subgroups SCT and Meihuashan, belonging to *P. t. amoyensis*, appeared to be clearly separated and high K values also differentiated other tiger subspecies (Figure 4C). When K = 10, all six living tiger subspecies were differentiated from each other and five clusters belonged to subgroups SCT and Meihuashan of the South China tigers (Figure 4C), except one tiger (#141) that might be a potential hybrid (similar with Wang et al. [14]). At this level, all individuals of Meihuashan were clearly divided into two groups (Figure 4). Out of the two groups, one group (M129, M130, and M131) had a closer relationship with pureblood SCTs than another group, which is supported by the NJ tree (Figure 4C) and PCA (Figure S12).

The three individuals (M129, M130, and M131) contained a proportion of Meihuashan that was less than 60%, shared a proportion of pureblood SCTs of more than 40% (Figure 4 and Table S6), and were analyzed to investigate their deleterious mutations to explore their potential as immigrants into pureblood SCTs. The number of nsSNPs (nonsynonymous SNPS) in M129 was 6797 (stopgain was 46) with 23.9% in a homozygous state, 6517 in M130 (stopgain was 53) with 22.7% in a homozygous state, and 6877 in M131(stopgain was 50) with 26.1% in a homozygous state. The number of homozygous sites with high-impact in the three individuals ranged from 6 to 11 and those with moderate-impact mutations from 520 to 752 (Table S7). Among the three individuals, M130 had the least number of homozygous sites for deleterious mutations with high and moderate impact (Table S7).

## 4. Discussion

Our results show that three hybrid individuals (M129, M130, and M131) share a proportion of pureblood SCT genetic materials amounting to more than 40% (Figure 4 and Table S6); they were clustered with pureblood SCTs in PCA for PC1 combined with PC2 (Figure S13) or PC1 combined with PC3 (Figure 4B), which is supported by the NJ tree (Figure 4C). So, the three individuals have more genome similarity with the pureblood SCTs than other hybrid individuals and are the candidates for genetically rescuing the SCT population.

Our results show the pureblood SCTs harbor a moderate level of microsatellite heterozygosity and nucleotide diversity, which is consistent with Zhang et al. [10] and Wang et al. [14]. Moreover, a large-scale genetic survey based on 319 pureblood SCTs with microsatellite data showed 82% (260) of 319 pureblood SCTs had low inbreeding (*f*_M_ < 0.125), which is comparable with that of giant pandas. Shan et al. [44] found that 78.3% (188) of the 240 sampled giant pandas, 65.2% (30) of the 46 wild-born giant pandas, and 81.4% (158) of the 194 captive-born giant pandas have an estimated inbreeding coefficient of *f*_M_ < 0.125. Except for careful genetic management [18], the low inbreeding in pureblood SCTs mainly comes from the introduction of #140 of the Chongqing line into the Suzhou line in 1995 [10], which caused the mean *f*_M_ to decrease significantly from 0.1789 to 0.0600 and the ratio of heterozygous loci to increase significantly from 38.5% to 43.2% (Figure 2).

However, the captive SCTs still suffer from inbreeding—30.4% (7/23) of the pureblood SCTs had long ROH lengths (>10 Mb) (Figure 3), which is probably the result of recent inbreeding [45], and 84% of the Wright inbreeding coefficient is due to recent inbreeding. Moreover, about 11% of microsatellite alleles were lost from the living pureblood SCTs. Some implications of inbreeding depression exist in the SCTs [5,12,17,18]; thus, some researchers [13,17] have suggested that the SCTs should be ‘rescued’ via genetic contributions from immigrant conspecifics or other subspecies to improve genetic diversity and decrease inbreeding among pureblood SCTs. However, Teixeira and Huber [46] demonstrated that no simple general relationship exists between neutral genetic diversity and the risk of species extinction. When populations are destined to remain small and isolated with high levels of inbreeding, management strategies should aim to minimize strongly deleterious variation rather than maximize genetic diversity [47]. Kuderna et al. [48] found within-species genetic diversity across families and geographic regions to be associated with climate and sociality but not with extinction risk. Thus, additional caution needs to be introduced into the current genetic rescue paradigm for pureblood SCTs [49]. Moreover, inbreeding depression is predominantly caused by the cumulative effects of deleterious mutations [50]; thus, we should check the genomes of all SCTs in the future in order to choose the individuals who are free from deleterious mutations to maintain the pureblood SCT population.

Considering the issue of inbreeding and the associated inbreeding depression among the current purebred SCTs, an alternative genetic management strategy is needed to establish a hybrid SCT population (denoted with back-up) based on the three individuals (M129, M130, and M131) to decrease the proportion of genetic materials from other tiger subspecies. However, a small tiger population is unstable and vulnerable—Miquelle et al. [51] reported a case about Amur tiger population in Sikhote-Alin Biosphere Zapovednik, where a population of 3–4 individuals recolonized in 1996, reached a peak of 38 in 2005, then rapidly dropped to less than 10 in 2012. Moreover, inbreeding depression is a particular concern in small, isolated populations, where the level of inbreeding will slowly increase and recessive deleterious mutations will become homozygous [22,47]. So, it is necessary to let a few immigrants from the pureblood SCTs into the back-up population in order to maintain its genetic diversity and increase the proportion of genetics of pureblood SCTs in this hybrid population. The present pureblood SCT population is large enough to introduce one or two individuals into the back-up population every year. The back-up population should be managed separately as an important reserve in case the pureblood SCT are in danger in the future.

## 5. Conclusions

The results of this study indicate that the current SCTs keep a moderate level of microsatellite heterozygosity and nucleotide diversity. However, the current SCTs still suffered from inbreeding. In order to decrease the extinction risk in the SCT population in the future, it is important to establish a back-up population based on the three individuals who had a closer relationship with pureblood SCTs than other hybrid individuals from Meihuashan through introducing one pureblood SCT into the back-up population every year.

## Figures and Tables

**Table 1:** The samples for living pureblood SCTs in this study.

Location	Living SCTs Numbers	Living SCTs Numbers for Microsatellite	Living SCTs Numbers for Genome
Canton Zoological Garden	14	14 (100%)	4
Changsha Zoological Garden	12	10 (83%)	0
Chengchou Zoological Garden	2	2 (100%)	0
Chengdu Zoological Garden	2	2 (100%)	0
Chongqing Zoological Garden	5	4 (80%)	0
Fuzhou Zoological Garden	1	1 (100%)	0
Hangzhou Wild Animal Park	17	17 (100%)	0
Kueiyang Qianling Park	1	1 (100%)	0
Laohu Valley Reserve Africa	18	1 (6%)	0
Linyi Botanical Garden	1	1 (100%)	0
Luoyang Wangcheng Park Zoo	57	56 (98%)	1
Meihuashan Natural Reserve	9	9 (100%)	0
Nanchang Zoological Garden	44	40 (91%)	6
Panyu Xiangjiang Safari Park	5	5 (100%)	0
Shanghai Zoological Garden	30	23 (77%)	3
Shaoguan Zoological Garden	11	10 (91%)	0
Suchou Zoological Garden	17	15 (88%)	0
Total	246	211 (86%)	14 (6%)

## Data Availability

All data generated or analyzed during this study are included in this published article and its supplementary information files. The raw sequencing reads from this study have been deposited into the CNGB Sequence Archive (CNSA) of China National GeneBank DataBase (CNGBdb) with accession number CNP0005449.

## Supplementary Materials

The following supporting information can be downloaded at: https://www.mdpi.com/article/10.3390/genes15040398/s1, Figure S1: The K value of Bayesian Clustering Analyses through software STRUCTURE (Usepopinfo = 1); admixture model and burn-in and replication values set at 50,000 and 100,000, respectively. The figure is based on all 320 captive South China tigers and uses 108 voucher tigers [2,15] as the reference tiger population data set. The figure shows K = 15 is the highest probability among choices of K. Figure S2: The Pearson correlation between *f*_M_ and *f*_P_. Figure S3: The Pearson correlation between the heterozygous loci ratio and *f*_P_. Figure S4: The Pearson correlation between the heterozygous loci ratio and *f*_M_. Figure S5: The Pearson correlation between *f*_P_ and generation. Figure S6: The Pearson correlation between *f*_M_ and generation. Figure S7: The Pearson correlation between the heterozygous loci ratio and generation. Figure S8: The distribution of *f*_M_ among the institutes with more than 3 living pureblood SCTs. The differences among locations ware not significant with *p* > 0.05 (ANOVA test). Figure S9: The Pearson correlation between heterozygosity and nucleotide diversity (π) at the genome level. Figure S10: The Pearson correlation between heterozygosity and *F*_ROH_ at the genome level. Figure S11: The comparation of *F*_ROH_ for total ROH between hybrids in Meihua mountain and pureblood SCTs (*p* = 0.0026, ANOVA). Figure S12: A comparation of *F*_ROH_ for different ROH lengths between hybrids in Meihua mountain and pureblood SCTs. (A) the ROH length < 0.1 M (*p* = 0.0001205, ANOVA); (B) 0.1 M ≤ the ROH length < 1 M (*p* = 0.001006, ANOVA); (C) 1 M ≤ the ROH length < 3 M (*p* = 0.0001264, ANOVA); (D) 3 M ≤ the ROH length < 5 M (*p* = 0.09333, ANOVA); (E) 5 M ≤ the ROH length < 10 M (*p* = 0.6082, ANOVA); (F) 10 M ≤ the ROH length (*p* = 0.0002751, ANOVA). Figure S13: The principal component analysis of PC1 and PC2 following autosomal variants. South China tigers include SCT and Mei. SCT denotes the 23 pureblood SCTs and Mei denotes the hybrid SCTs in Meihua mountain. ptal: *P. t. altaica*, ptco: *P. t. corbetti*, ptja: *P. t. jacksoni*, ptsu: *P. t. sumatrae*, and ptti: *P. t. tigris*. Table S1: The sample information in this study Table S2: Structure results based on microsatellite. Table S3: Re-sequencing data statistics in this study. Table S4: the genomic heterozygosity and *F*_ROH_ in this study. Table S5: the *F*_ROH_ under different ROH lengths. Table S6: The details about ADMIXTURE under genome level for the living six tiger subspecies. K is set from 4 to 10. Table S7: Individual homozygote SNP counts per impact category.
