# Supplementary material for: Insights for the Captive Management of South China Tigers Based on a Large-Scale Genetic Survey
Source: Genes (Basel). 2024 Mar 24;15(4):398. doi: 10.3390/genes15040398 (PMC11049310; doi:10.3390/genes15040398)
Supplement: Supplementary file 1 [file genes-15-00398-s001.zip › suplimentary material-20240215.pdf]

Supplementary material:

Insights for the Captive Management of South China Tigers Basing on Large-Scale Genetic Survey

Supplementary figure legend:

Figure S1: The K value of Bayesian Clustering Analyses through software STRUCTURE (Usepopinfo = 1), admixture model, and burn-in and replication values set at 50000 and 100000, respectively. The figure was based on all 320 captive South China tigers and the 108 voucher tigers (Luo et al. 2004 and 2008) as the reference tiger population data set. The figure showed K = 15 is the highest probability among choices of K.

Figure S2: The Pearson correlation between f_M and f_P.

Figure S3: The Pearson correlation between heterozygous loci ratio and f_P.

Figure S4: The Pearson correlation between heterozygous loci ratio and f_M.

Figure S5: The Pearson correlation between f_P and generation.

Figure S6: The Pearson correlation between f_M and generation.

Figure S7: The Pearson correlation between heterozygous loci ratio and generation.

Figure S8: The distribution of f_M among the institutes with more than 3 living pureblood SCTs. The differences among locations were not significant with $P > 0.05$ (ANOVA test).

Figure S9: The Pearson correlation between heterozygosity and nucleotide diversity (π) at genome level.

Figure S10: The Pearson correlation between heterozygosity and F_{ROH} at genome level.

Figure S11: The comparation of F_{ROH} for total ROH between hybridization in Meihua mountain and pureblood SCTs ($P = 0.0026$, ANOVA).

Figure S12: The comparation of F_{ROH} for different ROH length between hybridization in Meihua mountain and pureblood SCTs. (A) the ROH length < 0.1M ($P = 0.0001205$, ANOVA); (B) 0.1M ≤ the ROH length < 1M ($P = 0.001006$, ANOVA); (C) 1M ≤ the ROH length < 3M ($P = 0.0001264$, ANOVA); (D) 3M ≤ the ROH length < 5M ($P = 0.09333$, ANOVA); (E) 5M ≤ the ROH length < 10M ($P = 0.6082$, ANOVA); (F) 10M ≤ the ROH length ($P = 0.0002751$, ANOVA).

Figure S13 The principal component analysis of PC1 and PC2 following autosomal variants. South China tigers included SCT and Mei. SCT denoted the 23 pureblood SCTs and Mei denoted the hybridization SCTs in Meihua mountain. ptal: *P. t. altaica*, ptco: *P. t. corbetti*, ptja: *P. t. jacksoni*, ptsu: *P. t. sumatrae*, ptti: *P. t. tigris*.

Supplementary table legend

Table S1. The samples information in this study. (a separated file)

Table S2. Structure results basing on microsatellite. (a separated file)

Table S3. Re-sequencing data statistics in this study.

Table S4 the genomic heterozygosity and F_{ROH} in this study

Table S5 the F_{ROH} under different ROH length (a separated file)

Table S6. the detail about ADMIXTURE under genome level for the living six tiger subspecies. K is set from 4 to 10. (a separated file)

Table S7 Individual homozygote SNP counts per impact category.

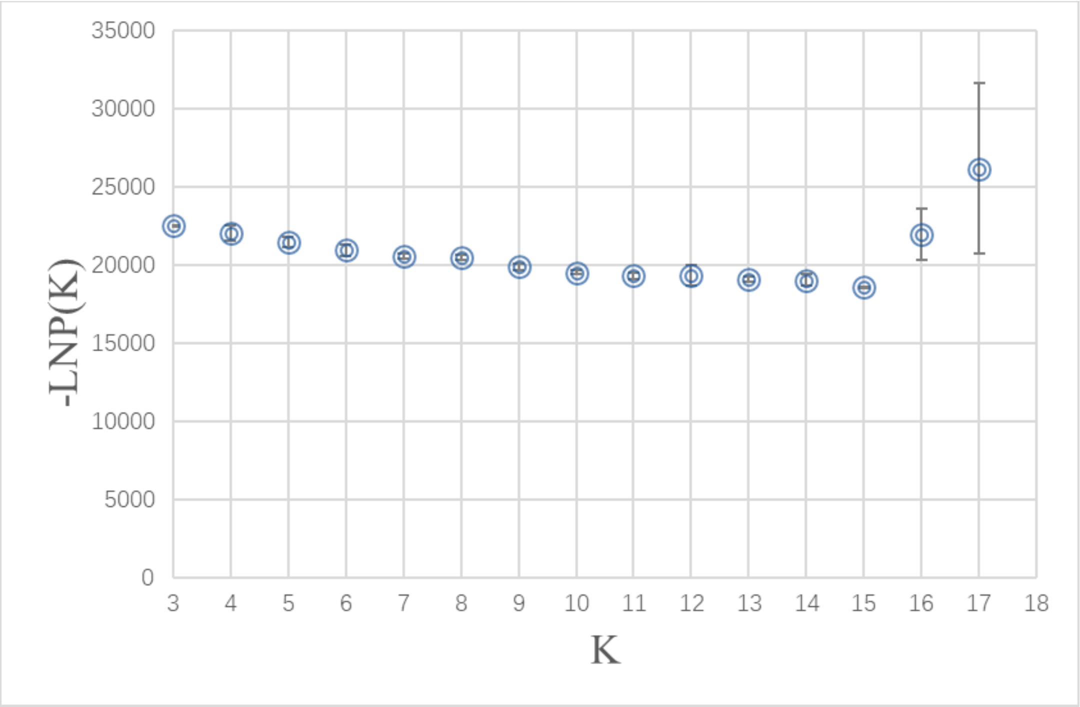

Figure S1: The K value of Bayesian Clustering Analyses through software STRUCTURE (Usepopinfo = 1), admixture model, and burn-in and replication values set at 50000 and 100000, respectively. The figure was based on all 320 captive South China tigers and the 108 voucher tigers (Luo et al. 2004 and 2008) as the reference tiger population data set. The figure showed K = 15 is the highest probability among choices of K.

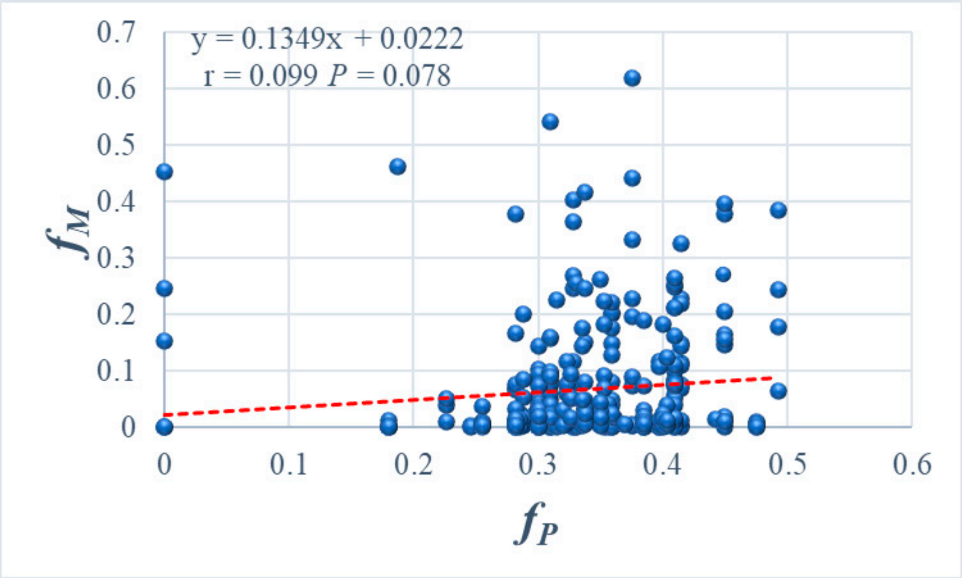

Figure S2: The Pearson correlation between f_M and f_P.

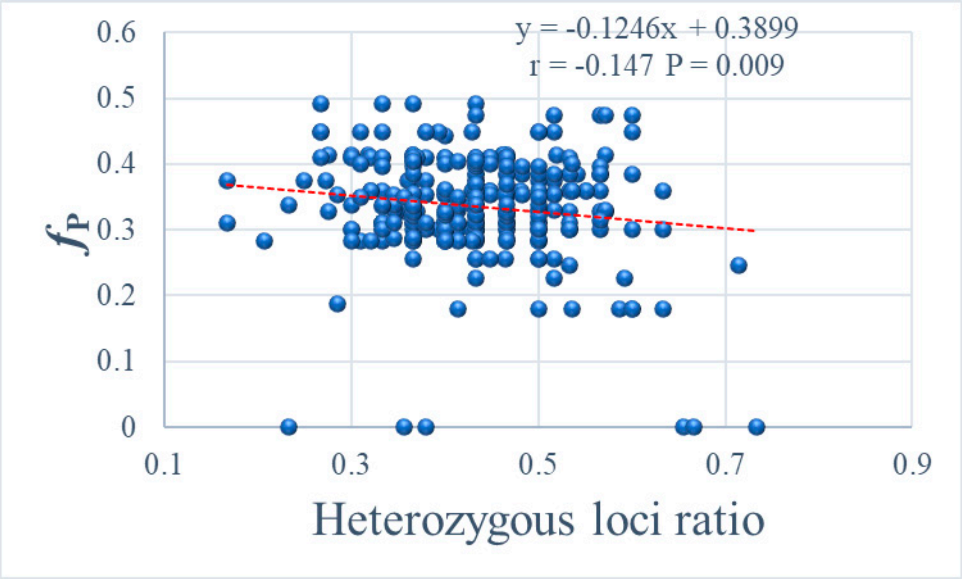

Figure S3: The Pearson correlation between heterozygous loci ratio and f_P.

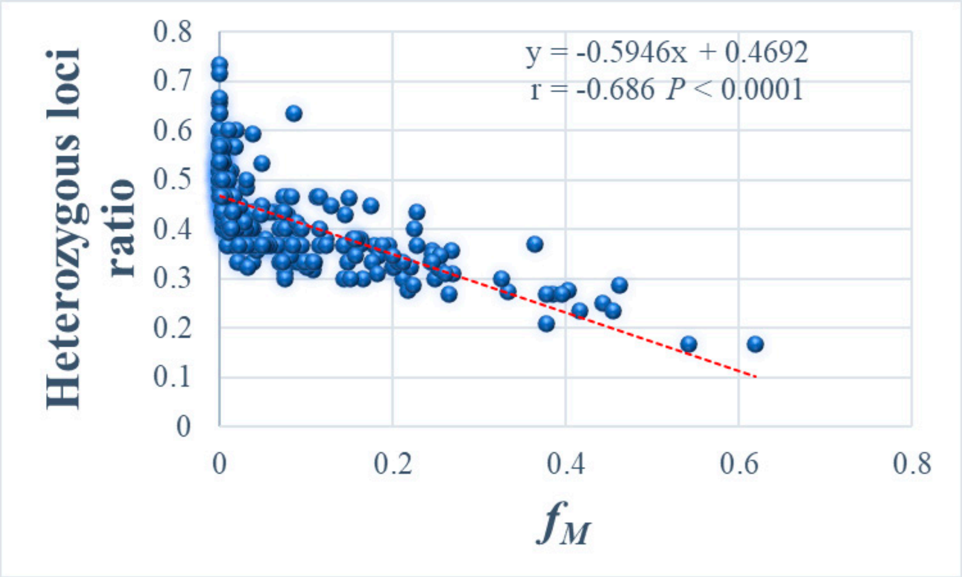

Figure S4: The Pearson correlation between heterozygous loci ratio and f_M.

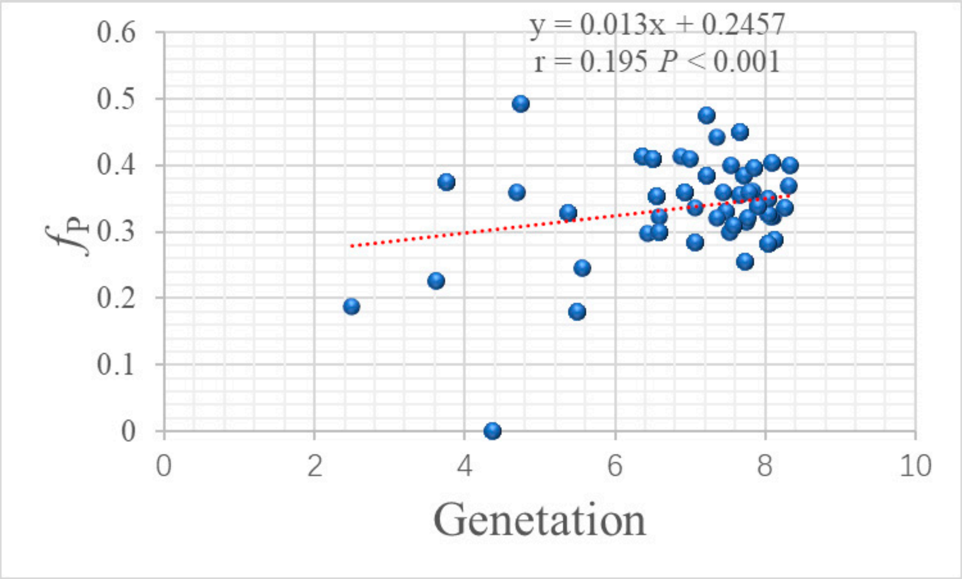

Figure S5: The Pearson correlation between f_P and generation.

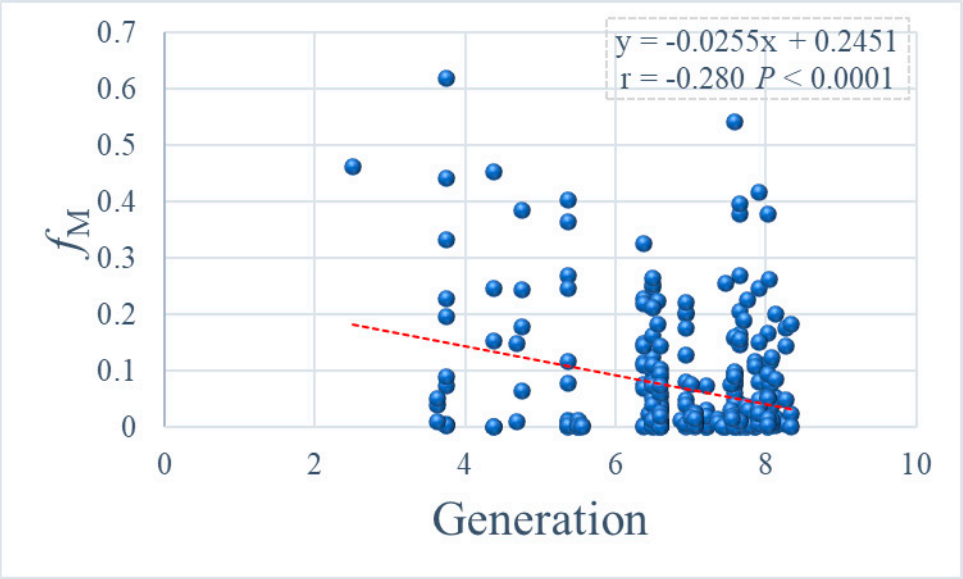

Figure S6: The Pearson correlation between f_M and generation.

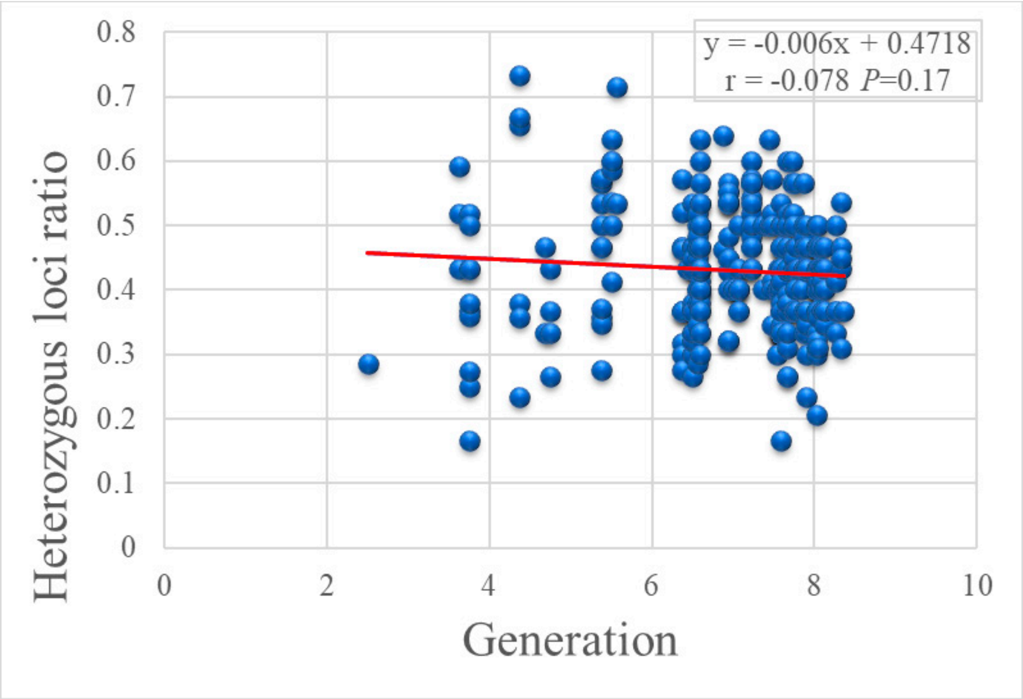

Figure S7: The Pearson correlation between heterozygous loci ratio and generation.

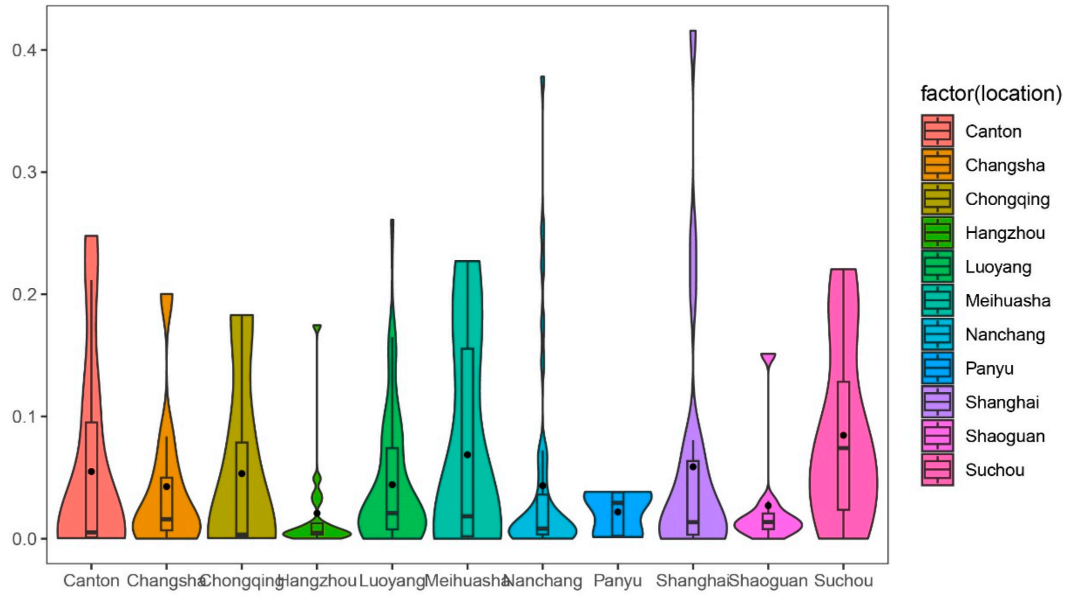

Figure S8: The distribution of f_M among the institutes with more than 3 living pureblood SCTs. The differences among locations were not significant with $P > 0.05$ (ANOVA test).

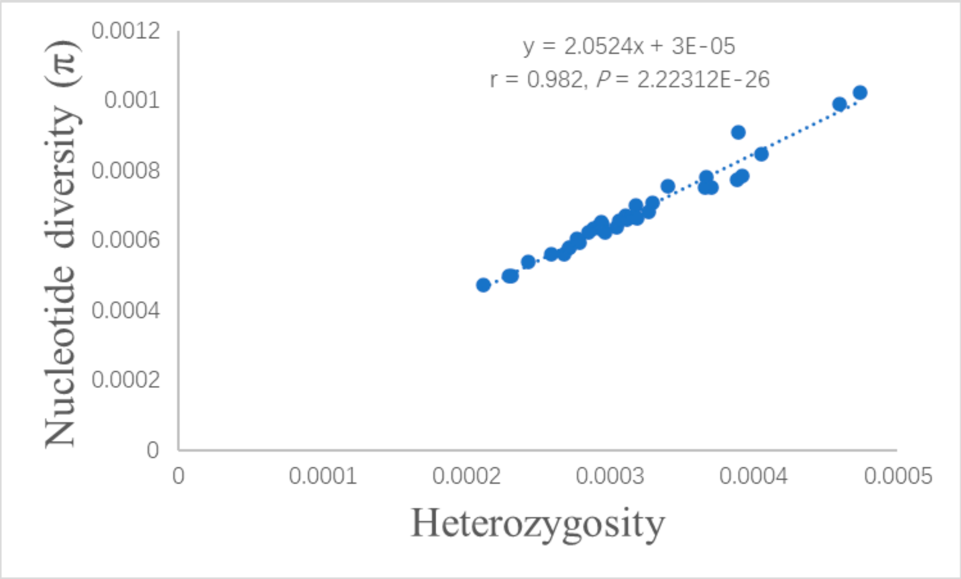

Figure S9: The Pearson correlation between heterozygosity and nucleotide diversity (π) at genome level.

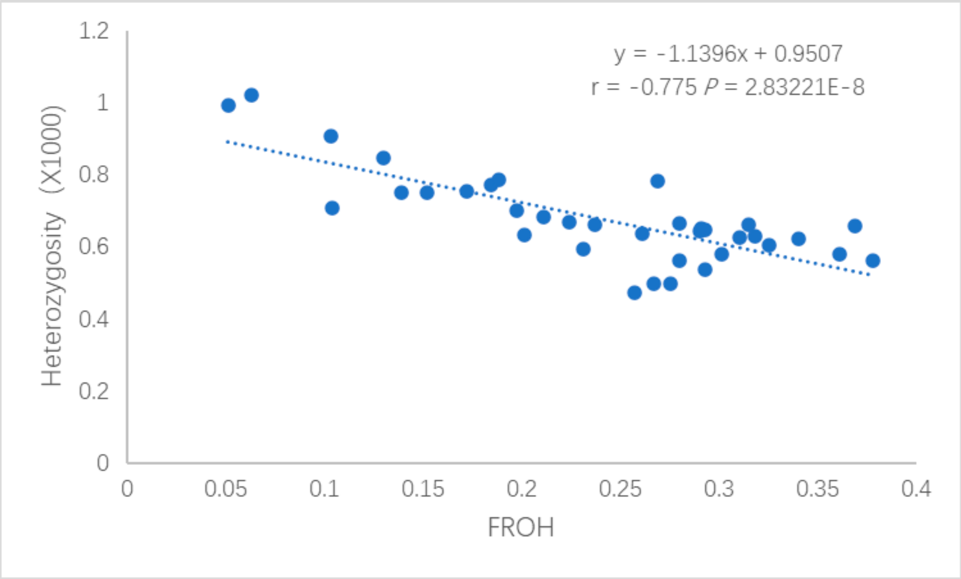

Figure S10: The Pearson correlation between heterozygosity and F_{ROH} at genome level.

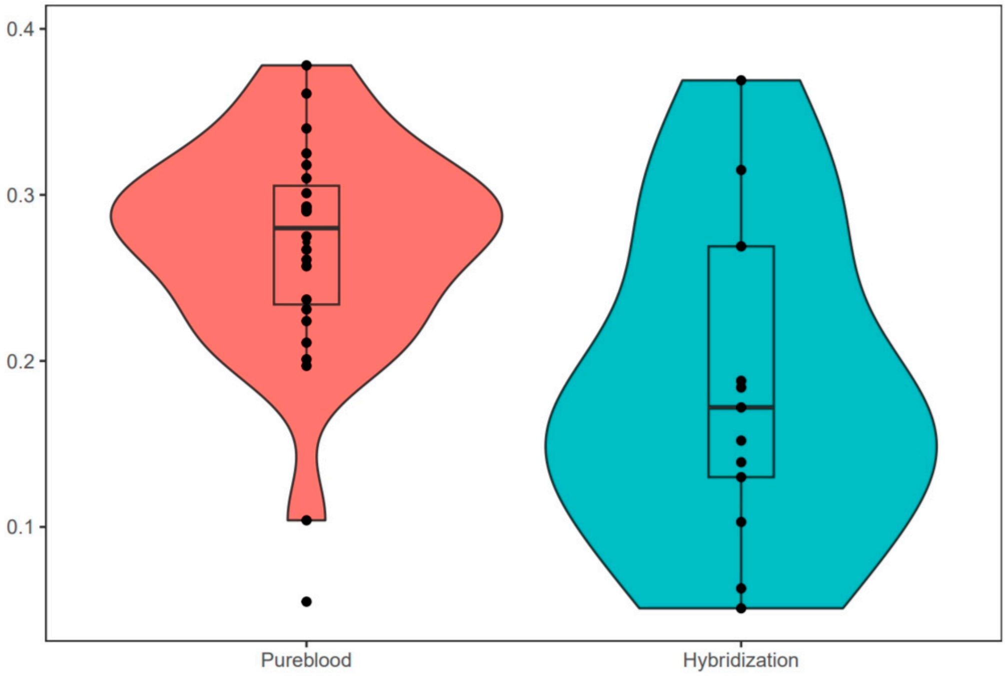

Figure S11: The comparation of F_{ROH} for total ROH between hybridization in Meihua mountain and pureblood SCTs (P = 0.0026, ANOVA).

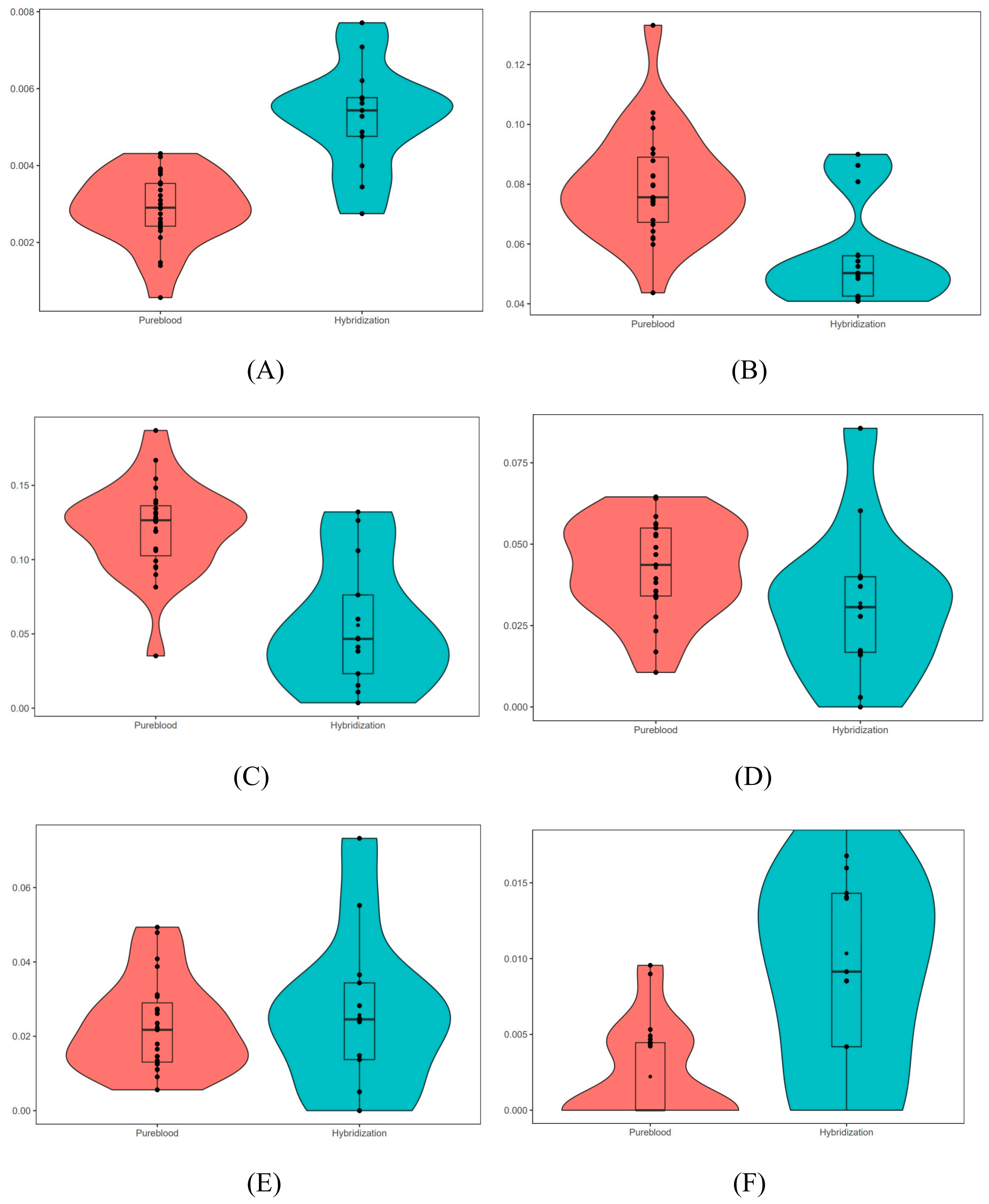

Figure S12: The comparation of F_{ROH} for different ROH length between hybridization in Meihua mountain and pureblood SCTs. (A) the ROH length < 0.1M (P = 0.0001205, ANOVA); (B) 0.1M ≤ the ROH length < 1M (P = 0.001006, ANOVA); (C) 1M ≤ the ROH length < 3M (P = 0.0001264, ANOVA); (D) 3M ≤ the ROH length < 5M (P = 0.09333, ANOVA); (E) 5M ≤ the ROH length < 10M (P = 0.6082, ANOVA); (F) 10M ≤ the ROH length (P = 0.0002751, ANOVA).

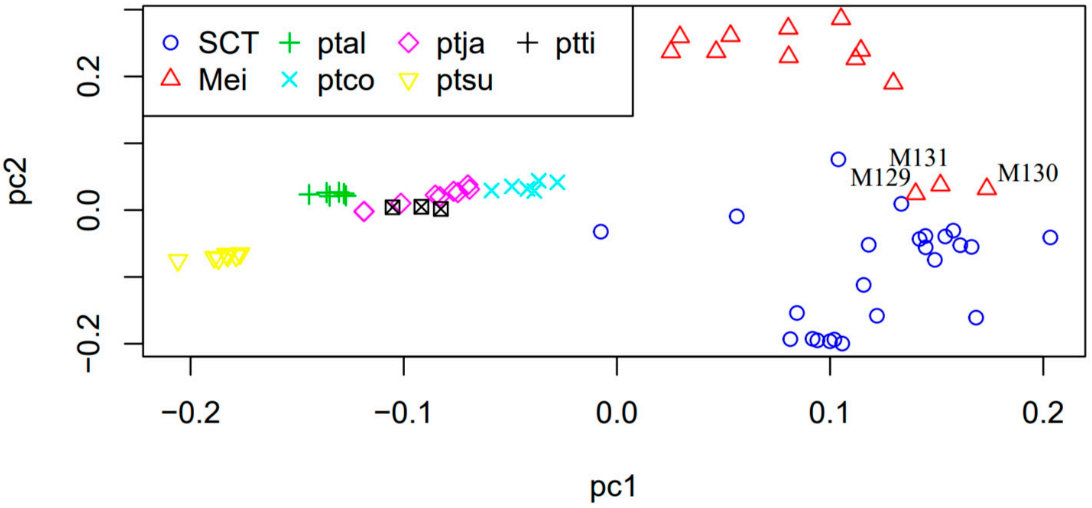

Figure S13 The principal component analysis of PC1 and PC2 following autosomal variants. South China tigers included SCT and Mei. SCT denoted the 23 pureblood SCTs and Mei denoted the hybridization SCTs in Meihua mountain. ptal: *P. t. altaica*, ptco: *P. t. corbetti*, ptja: *P. t. jacksoni*, ptsu: *P. t. sumatrae*, ptti: *P. t. tigris*.

Table S1. The samples information in this study. (a separated file)
Table S2. Structure results basing on microsatellite. (a separated file)

Table S3. Re-sequencing data statistics in this study.

Sample	Read_length(bp)	RawBases(Gb)	RawCoverage(X)	CleanBases(Gb)	CleanCoverage(X)
#267	150_150	49.90826	20.8	47.94401278	19.98
#296	150_150	44.61216	18.59	43.0103104	17.92
#299	150_150	39.07528	16.27	37.50236	15.62
#348	150_150	37.65111	15.67	36.254522	15.1
#393	150_150	38.72085	16.12	36.747563	15.29
#441	150_150	46.13822	19.21	43.827861	18.25
#457	150_150	34.10595	14.2	32.957172	13.73
#467	150_150	37.90963	15.78	36.580036	15.23
#468	150_150	44.13163	18.38	41.101204	17.11
#489	150_150	44.93985	18.71	42.607143	17.73
#522	150_150	40.3453	16.8	38.747885	16.13
#530	150_150	41.69762	17.36	39.582499	16.47
#531	150_150	41.50857	17.27	39.477911	16.43
#544	150_150	34.67444	14.43	33.165348	13.81
#551	150_150	47.24652	19.67	44.92909	18.71
#553	150_150	46.49082	19.36	44.311691	18.45
#558	150_150	48.53714	20.2	46.351285	19.3
#616	150_150	41.2668	17.18	39.281961	16.35
#625	150_150	44.37436	18.47	42.410637	17.66
#635	150_150	35.00888	14.57	33.61567	14
#701	150_150	42.69144	17.77	40.710288	16.95
#702	150_150	43.09487	17.94	41.207888	17.15
#712	150_150	32.83035	13.67	31.538613	13.13
M118	150_150	44.48234	18.53	43.886367	18.27
M129	150_150	47.38267	19.74	39.466503	16.43
M130	150_150	43.25693	18.02	40.379301	16.81
M131	150_150	51.26582	21.36	42.877485	17.85
M29	150_150	45.30731	18.88	43.819113	18.24
M31	150_150	48.864211	20.36	46.599321	19.4
M36	150_150	43.080932	17.95	41.194761	17.15
M44	150_150	45.5760134	18.99	43.428646	18.08
M64	150_150	45.192257	18.83	43.236483	18
M67	150_150	40.968016	17.07	39.393241	16.4
M70	150_150	46.93199	19.56	44.768306	18.65

M74	150_150	52.45002	21.85	50.145154	20.89
M77	150_150	43.17973	17.99	41.141724	17.14

Table S4 the genomic heterozygosity and F_{ROH} in this study

Individual	Total ROH length (Kb)	the number of ROH	Average ROH length (Kb)	Number of heterozygotes	F_{ROH}	non-ROH	Heterozygosity	N_SITES	Covered(bp)	CoveredRatio(%)	Origin
#267	693753	739	938.772	1539512	0.29	0.71	0.000643078	2376436	2393974405	99.1	pureblood
#296	905833	895	1012.1	1342943	0.378	0.622	0.00056107	2367254	2393537867	99.08	pureblood
#299	478368	613	780.372	1504520	0.201	0.799	0.000633491	2009701	2374965919	98.31	pureblood
#441	856086	961	890.828	1373068	0.361	0.639	0.000578736	2257874	2372530894	98.21	pureblood
#530	694938	816	851.639	1533596	0.293	0.707	0.000646195	2415206	2373270732	98.24	pureblood
#551	807383	999	808.191	1480910	0.34	0.66	0.000623663	2444966	2374537488	98.3	pureblood
#616	619749	726	853.648	1516877	0.261	0.739	0.000638408	2189493	2376028907	98.36	pureblood
#701	530718	717	740.193	1589963	0.224	0.776	0.000669779	2259842	2373861531	98.27	pureblood
#702	563601	739	762.654	1575202	0.237	0.763	0.000663101	2249524	2375507455	98.34	pureblood
#348	246572	448	550.383	1684277	0.104	0.896	0.000708491	2069506	2377275259	98.41	pureblood
#393	664980	768	865.85	1584009	0.28	0.72	0.000666514	237556	2376556699	98.38	pureblood

			9					3			
#457	610577	520	1174.19	1118968	0.257	0.743	0.000471408	1518812	2373672443	98.26	pureblood
#467	652237	686	950.783	1184568	0.275	0.725	0.000498584	1701982	2375862752	98.35	pureblood
#468	632885	621	1019.14	1183372	0.267	0.733	0.000498352	1672940	2374570114	98.3	pureblood
#489	772474	845	914.17	1435047	0.325	0.675	0.000604443	2328429	2374162803	98.28	pureblood
#522	467853	824	567.783	1661572	0.197	0.803	0.000699608	2277258	2375003370	98.32	pureblood
#531	755879	929	813.648	1496392	0.318	0.682	0.000630478	2413959	2373424450	98.25	pureblood
#544	695086	823	844.576	1278600	0.293	0.707	0.00053857	1929810	2374064437	98.28	pureblood
#553	691404	948	729.33	1549346	0.291	0.709	0.000652548	2424024	2374300162	98.29	pureblood
#558	715804	838	854.181	1377323	0.301	0.699	0.000579561	2101549	2376493772	98.38	pureblood
#625	500523	667	750.409	1623129	0.211	0.789	0.000682882	2252058	2376881704	98.39	pureblood
#635	735365	1185	620.561	1484502	0.31	0.69	0.000624977	2352420	2375289131	98.33	pureblood
#712	548027	620	883.915	1410043	0.231	0.769	0.000593776	1939973	2374703827	98.3	pureblood
M29	151309	729	207.557	2450524	0.063	0.937	0.001023128	3288507	2395129796	99.15	hybridization
M31	120311	661	182.013	2355704	0.051	0.949	0.000991829	3091882	2375111375	98.32	hybridization
M3	223962	72	309	19832	0.	0.89	0.0009	283	21835	90.39	hybri

6		3	.768	93	103	7	0829	5224	46758		dization
M44	310632	648	479.37	2029831	0.13	0.87	0.000846703	2812730	2397335770	99.24	hybridization
M64	440750	665	662.782	1855134	0.184	0.816	0.000773832	2646703	2397335770	99.24	hybridization
M67	451466	760	594.034	1883845	0.188	0.812	0.000785333	2766137	2398785187	99.3	hybridization
M70	644717	960	671.58	1872865	0.269	0.731	0.000781398	2919683	2396812652	99.22	hybridization
M74	754580	947	796.811	1582520	0.315	0.685	0.000660159	2634908	2397180527	99.23	hybridization
M77	883849	1013	872.507	1573854	0.369	0.631	0.000656754	2762271	2396413501	99.2	hybridization
M118	634363	736	861.906	1272569	0.28	0.72	0.000561193	2195728	2267612945	93.87	hybridization
M129	362488	570	635.944	1786477	0.152	0.848	0.000750562	2430861	2380184335	98.53	hybridization
M130	332556	497	669.127	1803346	0.139	0.861	0.000752305	2382585	2397094200	99.23	hybridization
M131	412917	630	655.424	1813423	0.172	0.828	0.000756204	2534285	2398060478	99.27	hybridization

Table S5 the F_{ROH} under different ROH length (a separated file)

Table S6. the detail about ADMIXTURE under genome level for the living six tiger subspecies. K is set from 4 to 10. (a separated file)

Table S7 Individual homozygote SNP counts per impact category.

Individual	High	Moderate	Low	Total
M129	10	625	989	1624
M130	6	520	951	1477
M131	11	752	1032	1795
